# Integration of haptic virtual reality simulators in undergraduate dental curricula: A survey-based study in Gulf Cooperation Council countries

**DOI:** 10.1371/journal.pone.0322810

**Published:** 2025-05-28

**Authors:** Manal Matoug-Elwerfelli, Jumma Al-Khabuli, Hazza Alhobeira, Hanin Dass, Ahmed Abdou, Kamran Ali

**Affiliations:** 1 Department of Pre-Clinical Oral Sciences, College of Dental Medicine, QU Health, Qatar University, Doha, Qatar; 2 Department of Restorative Dentistry, College of Dentistry, University of Hail, Hail, Kingdom of Saudi Arabia; 3 Department of Restorative Dentistry, Faculty of Dentistry, University of Malaya, Kuala Lumpur, Malaysia; A T Still University Missouri School of Dentistry & Oral Health, UNITED STATES OF AMERICA

## Abstract

**Background:**

The integration of haptic simulators in contemporary dental education has been reported to improve students’ hand-eye coordination and fine motor skills during pre-clinical education to facilitate a smooth transition to the clinical setting. The aim of this study was to assess the integration of haptic virtual reality simulation (HVRS) in undergraduate dental curricula in the Gulf Cooperation Council countries.

**Methods:**

All dental schools offering undergraduate dental programs in the Gulf Cooperation Council countries were invited to participate in this cross-sectional study design. Data was collected using an online survey on a voluntary basis and analyzed using Microsoft Excel.

**Results:**

Out of 34 dental schools, responses were received from 30 dental schools (response rate 88.2%). In terms of haptic integration, only two (6.7%) dental institutions have adopted haptic simulation in undergraduate dental education. However, a considerable proportion of schools (n = 13, 46.4%) expressed an interest in the future use of haptic technology. The key strengths of HRVS included the integration of modern technology, opportunities for self-directed learning, development and consolidation of manual skills, and boosting self-confidence amongst undergraduate dental students. Financial cost and limited patient cases in the HRVS library were regarded as the main barriers to widespread use of this technology.

**Conclusion:**

Although the Gulf Cooperation Council countries have strong economies with a high gross domestic product (GDP), only a limited number of dental schools have incorporated haptic technology in their curricula. Nevertheless, a high proportion of dental schools in the region are actively considering purchasing and implementation of haptic devices in undergraduate dental programs.

## Introduction

The integration of new technologies in dental education is essential to the learning experiences of dental students and preparing them for contemporary clinical practice. Digital tools such as virtual reality (VR) simulation, artificial intelligence (AI)-assisted diagnostics, and computer-aided design/computer-aided manufacturing (CAD/CAM) systems provide students with hands-on, interactive learning opportunities that improve skill acquisition and decision-making. By incorporating these innovations, dental education can bridge the gap between theoretical knowledge and practical application, ultimately leading to more competent and confident dental professionals. Undergraduate dental students are required to demonstrate competency in a wide range of invasive and irreversible procedures under supervision [1,2].

Developing practical skills can be challenging and stressful for dental students and require structured training to ensure patient safety. Dental institutions must ensure that students develop core practical skills before they are allowed to provide clinical interventions on real patients. Pre-clinical training is essential for a smooth transition of dental students into the clinical settings, development of clinical competence, and to reduce the risks of clinical mishaps [3]. Traditional dental teaching and learning activities in a pre-clinical skills laboratory involve artificial and/or extracted human teeth mounted in models and phantom heads (manikin-based simulators) [4]. Although, these methods are well-established and suitable for supporting the learning activities of dental undergraduate students, limitations such as the maintenance and regulation of infection control procedures, and the cost and consumption of multiple materials and consumables are recognized [4]. Moreover, simulated dental learning on mannequins requires close supervision of novice undergraduate students, which has cost implications due to the use of consumables, and provision of human resources.

VR is as a computer-generated simulation of a real life situation/environment and provides an immersive learning experience [5,6]. The integration of VR simulators in contemporary dental curricula aims to improve students’ hand-eye coordination and fine motor skills in pre-clinical settings to ensure a smooth transition to the clinical setting [7]. Modern dental VR simulators also offer haptic feedback to learners during dental operative procedures [8]. Haptic virtual reality simulation (HVRS) may provide a realistic learning experience to novice dental students in a non-threatening environment where students are able to visualize dental and oral anatomical structures and learn a range of operative dental procedures [9]. HVRS has been used effectively for training of medical students to learn a wide range of various procedures and tasks such as cannulation, injection administration, arthroscopy, endoscopy, and laparoscopy [10–13]. In Dentistry, HVRS can be used to provide practical training on developing manual dexterity, and learning a wide range of procedural skills in operative dentistry, endodontics, prosthodontics oral & maxillofacial surgery, pediatric dentistry [14–19].

There is a growing focus on evaluating the integration of HVRS in dental education and training to inform evidence-based educational strategies [20]. Published studies have reported that HVRS is an effective adjunct teaching tool, which can complement the traditional dental training on mannequins and physical models [4,9,21]. The reported benefits of HRVS include structured simulated learning to develop basic skills, automated and immediate student feedback, and reduced reliance on supervision by the dental faculty [4,7,9]. Previous studies on integration of simulation technologies in dental curricula have largely focused on developed countries [22]. The aim of the current study was to assess the integration of HVRS within undergraduate dental curricula in the Gulf Cooperation Council (GCC) countries.

### Conceptual framework

The conceptual framework of this study was informed by the Universal Design for Learning [23,24]. This theory underscores the importance of designing learning experiences that are accessible to all learners, regardless of their abilities or learning styles. New technological advancements such as HVRS may be used to provide multiple modes of representation, expression, and engagement, which facilitate learners to access and benefit from modern technologies to develop practical skills.

## Methods

### Ethical approval

Ethical approval of this study was obtained from the Institutional Review Board (IRB) at Qatar University, State of Qatar (Approval number: QU-IRB 1652-EA/22). Informed consent was obtained from the participating participants via Google Forms prior to the questionnaire completion.

### Study design

An analytical cross-sectional study design was used to survey the target dental institutions.

### Sampling technique and recruitment

A non-randomized purposive sampling technique was used to target all dental institutions/universities that offer an undergraduate dental program in the GCC countries, namely Kingdom of Saudi Arabia (KSA), State of Kuwait, State of Qatar, Sultanate of Oman, and the United Arab Emirates. The Kingdom of Bahrain was excluded, as it does not offer an undergraduate dental program at present. Each institution was invited to participate in the study through a nominated representative responsible for overseeing pre-clinical undergraduate dental education in the respective institution. Invitations to participate in the study were sent using institutional emails. A participant information sheet explaining the purpose and scope of study and the contact details of the research team accompanied the email invitations.

### Data collection instrument

A team of experienced dental academics developed a purposefully designed questionnaire. Relevant literature was explored to identify the key dimensions of training of dental students in simulated dental laboratory settings. The questionnaire was adopted from a previous survey [22]. The draft questionnaire was pre-tested to ensure that the language of questions was clear, and unambiguous and amenable to interpretation by the participants. Ten independent dental academics were invited to review the questionnaire to assess the content, language clarity, response categories and identify any ambiguities, duplication or potential errors. Their responses for the pilot study were collated and analyzed by the research team and the questionnaire was updated in the light of feedback. The final questionnaire consisted of a combination of closed and open-ended items. The full survey questionnaire is included as an appendix (Supplementary material).

### Data collection

A total of 34 undergraduate dental institutions/universities within the GCC region were identified. A representative from each dental school was invited to complete an online questionnaire on a voluntary basis. This survey was conducted via Google Forms between 01/12/2022 and 28/2/2023, with a reminder email sent four weeks from the initial invite. Incomplete or multiple responses from the same institute were rejected/deleted.

### Data analysis

Responses to the online questionnaire were processed and analyzed using Microsoft Excel. All descriptive and frequency-based data were represented using tables and bar charts.

## Results

### Demographics

A total of 30 dental schools participated in the survey, with a response rate of 88.2%. The characteristics and geographic distribution of the participating institutions are presented in Table 1 and 2. The majority of dental schools in the region are classified as public/governmental (n = 19, 63.3%) and private schools (n = 10, 33.3%). Most schools in the region award either a 6-year Bachelor of Dental Surgery (n = 18, 60%) or a 5-year Bachelor of Dental Surgery (n = 7, 23.3%). The most common cohort sizes of dental students per academic year in participating institutions was 26–50 students (n = 9, 30%), 1–25 students (n = 7, 23.3%) or 51–75 students (n = 7, 23.3%).

**Table 1:** Characteristics of participating dental institutions.

Category	Subcategory	Frequency (n)	Percentage
**School Type**	Public/Governmental	19	63.3%
	Private	10	33.3%
	Hybrid	1	3.3%
**Degree Awarded**	5-year Bachelor of Dental Surgery (BDS)	7	23.3%
	6-year Bachelor of Dental Surgery (BDS)	18	60.0%
	6-year Doctor of Dental Medicine (DDM)	2	6.7%
	6-year Doctor of Dental Surgery (DDS)	2	6.7%
	7-year Dual Degree (DDM & BMedSci)	1	3.3%
**Cohort Size per Academic Year**	1-25 students	7	23.3%
	26-50 students	9	30.0%
	51-75 students	7	23.3%
	76-100 students	4	13.3%
	101-150 students	2	6.7%
	151-200 students	1	3.3%

**Table 2:** Distribution of the participating dental institutions.

	Type of dental institution/university			Total Responses
	Public (Government)	Private	Hybrid	
**Kingdom of Saudi Arabia**	17	6	0	23
**Kuwait**	1	0	0	1
**Oman**	0	1	0	1
**Qatar**	1	0	0	1
**United Arab Emirates**	0	3	1	4

### Implementation, perceived benefits and challenges of HVRS usage

A large majority of the dental institutions in GCC countries did not use HVRS technology in undergraduate dental education (n = 28, 93.3%). Nevertheless, a large number of the participating institutions (n = 13, 46.4%) expressed an interest in the future use of HVRS. Only two (6.7%) of the participated dental schools in the GCC region currently use HVRS in the undergraduate dental education. Schools adopting HVRS technology included Ajman Dental College, UAE (VirTeaSy Dental) and Qatar University (SIMtoCARE Dente^®^).

Regarding the usage at various stages of the undergraduate program, training in manual dexterity and dental charting was seen in the early years of undergraduate dental education training (year 2) with a reported usage of 11–20 hours/course. In the third and fourth year of undergraduate training, courses implementing HVRS training included; conservative/operative dentistry, endodontics, periodontology and prosthodontics with the highest usage of HVRS (approximately 40 hours/course), in comparison to other dental courses. Courses such as oral surgery and pediatric courses were also integrated, although at a lower level with minimal contact hours of 1–10 hours/course. Both dental schools did not report HVRS usage in implantology or orthodontics at the time of data collection. In terms of evaluation of student learning following HVRS usage, the reliance on automated feedback, self-evaluation, and summative assessment were reported.

**Fig 1:**
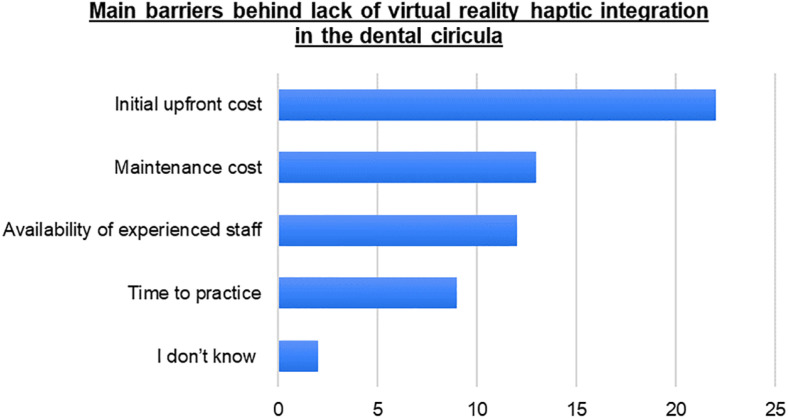
Bar chart to summarize key barriers to integrating haptic virtual reality simulators in undergraduate dental curricula.

Both dental schools implementing HVRS reported similar benefits and challenges of the use of HVRS on the learning experiences of students. Implementation of modern technology, self-directed learning, development and consolidation of manual skills (opportunities for repeated practice), boosting self-confidence, reduced need for supervision, and acquisition of technical skills were regarded as beneficial to the students. However, the maintenance cost, limited tasks within the library software, limited number of available VR units and lack of experienced staff were reported as the main challenges. In-line with the above, the remaining dental schools in the region whom do not use HVRS reported that the initial upfront cost (n = 22, 78.6%), maintenance cost (n = 13, 46.4%), lack of experienced staff (n = 12, 42.9%), limited time to practice (n = 9, 32.1%) as the main barriers behind the lack of integration of HVRS within the dental curriculum. These results are depicted in Fig 1.

## Discussion

Pre-clinical training in a simulated environment is a core component of preclinical dental education. It is essential to impart practical skills in a safe environment before students transition into clinical training where they are able to perform invasive and irreversible procedures on real patients [22,25]. While traditional mannequin-based simulation is widely regarded as an effective training method, the integration of digital technologies, such as HVRS, signifies evolution of dental education. HVRS offers several advantages, including self-paced learning and the ability to repeatedly practice standardized procedures with minimal supervision [22]. Unlike mannequin-based simulation, HVRS eliminates the need for physical dental instruments and materials, thereby reducing clinical waste production and minimizing the risk of sharps injuries to novice learners [17].

This study represents the first investigation into HVRS integration in undergraduate dental education within the GCC region. Despite the presence of well-established dental schools in Saudi Arabia, HVRS remains absent from the undergraduate curricula. The results show that in addition to financial cost, key barriers to incorporation of HVRS include a lack of experienced faculty (n = 12) and time limitations (n = 9). These challenges could be addressed through faculty training workshops, professional development programs, or the recruitment of experts in digital dental education.

The HVRS equipment at the College of Dentistry, Ajman University, UAE is VirTeaSy Dental (Laval, France). This institution represents the country’s first dental school, and was founded in 1997. Similarly, SIMtoCARE Dente^®^ (Vreeland, The Netherlands) is used at the College of Dentistry, Qatar University, the only dental school in Qatar established in 2019. A global comparison survey reveals that HVRS adoption varies across regions, with higher frequency of use in Asia (n = 18/24) and Oceania (n = 4/6) compared to Europe (n = 5/20) and North America (n = 2/12) [22]. However, these numbers require regular updates as a growing number of dental institutions are actively engaged in the provision of HVRS technology. Furthermore, the COVID-19 pandemic accelerated interest in remote education models with reduced face-to-face contact with faculty supervisors, further emphasizing the relevance of HVRS in contemporary dental training [26].

The results of this study indicate that HVRS is currently applied across multiple dental disciplines, including manual dexterity training, dental charting, operative dentistry, endodontics, prosthodontics, periodontics, and pediatric dentistry. Notably, manual dexterity and dental charting are introduced early in Year 1 and 2, while practical training on skills related to specialized disciplines such as operative dentistry, endodontics, and prosthodontics are provided during Years 3 and 4. It needs to be reiterated that the current study focused on the utilization of HVRS in GCC countries and did not evaluate improvements. This acknowledged as a limitation of the current study. Nevertheless, previous research provides evidence to support the use of HVRS for imparting manual skills and spatial perception in dental education, with a direct correlation to overall student performance [14,27].

Despite its broad applications, HVRS remains underutilized in oral surgery training, with only one institution (Ajman Dental College, UAE) reporting its use. However, the College of Dentistry at Qatar University is actively collaborating with manufacturers to develop a local anesthesia training module within HVRS library, which could enhance student competency in local anesthesia techniques. Previous studies have demonstrated the benefits of VR-based anesthesia training, reporting improved student engagement, anatomical understanding, and technical skill acquisition [28]. Moreover, recent reviews suggest that simulated VR environments provide valuable training for complex maxillofacial procedures, including dental implant placement and orthognathic surgery, potentially leading to improved surgical precision and patient outcomes [6,18,29]. These findings underscore the potential of HVRS in specialist postgraduate dental education programs.

Feedback and assessment are integral to student learning, self-reflection, and skill progression, and should be strategically incorporated into HVRS training [30]. The participating institutions in the current study reported employing various evaluation methods, including automated software feedback, self-assessment, and summative assessments. To maximize the educational impact of HVRS, assessment strategies should be tailored to align with the available software. However, further research is required to evaluate the appropriate feedback strategies for training based on HVRS.

One limitation frequently cited by institutions was the restricted number of clinical cases available within the HVRS software. However, collaborating with manufacturers to expand case libraries is feasible. Some advanced HVRS systems now offer the ability to import real patient data along with cone beam computed tomography scans allowing students to practice on case-specific virtual models. This enhancement provides a more realistic training experience, boosting student confidence and ensuring better preparedness for patient care [16,31]. Although HVRS significantly enhances preclinical training, it may not replace traditional hands-on experiences using dental mannequins and models. A blended approach, combining HVRS with mannequin-based simulation may be more appropriate to ensure the effective transfer of skills to clinical practice [27].

Results of this study highlights that initial cost of HVRS equipment remains a primary barrier to widespread adoption, aligning with findings in existing dental education literature [7]. However, the initial cost of HVRS may be off-set over time as it eliminates the need for physical models, disposable materials, and hazardous waste disposal [32]. Additionally, the reduced need for faculty supervision may further lower operational costs. Nevertheless hardware maintenances and software updates are still required [18].

This study captures data from a specific geographic region where HVRS adoption remains limited. Therefore, the findings may be interpreted with a degree of caution. Nonetheless, this research provides valuable insights into HVRS integration, highlighting its benefits and key challenges. Moving forward, it is essential to collaborate with industry stakeholders to address financial and logistical barriers, facilitating the broader adoption of HVRS in undergraduate dental curricula.

## Conclusions

The results of this survey provide an overview of HVRS integration in undergraduate dental curricula across the GCC region. Despite the relatively recent establishment of many dental schools and the region’s strong economic standing, HVRS adoption remains limited. Nevertheless, many institutions are actively considering future investments in this technology. There is a need to address barriers to a more widespread use HVRS such as faculty training, curriculum adaptation, and cost of equipment.

## Supporting information

S1 File Questionnaire. https://doi.org/10.6084/m9.figshare.28714658.v1.
(PDF)
