# Supplementary material for: Integration of haptic virtual reality simulators in undergraduate dental curricula: A survey-based study in Gulf Cooperation Council countries
Source: PLoS One. 2025 May 28;20(5):e0322810. doi: 10.1371/journal.pone.0322810 (PMC12118929; doi:10.1371/journal.pone.0322810)
Supplement: S1 File — https://doi.org/10.6084/m9.figshare.28714658.v1. (PDF) [file pone.0322810.s001.pdf]

Supplementary material

Integration of haptic virtual reality simulators in undergraduate dental curricula: A survey-based study in Gulf Cooperation Council Countries

Manal Matoug-Elwerfelli[1], Jumma Al-Khabuli[1], Hazza Alhobeira[2], Hanin Dass[1], Ahmed Abdou[3], Kamran Ali[1]

[1] College of Dental Medicine, QU Health, Qatar University, Doha, Qatar
[2] College of Dentistry, The University of Hail, Kingdom of Saudi Arabia
[3] Department of Restorative Dentistry, Faculty of Dentistry, University of Malaya, Kuala Lumpur, Malaysia

Corresponding author: Manal Matoug-Elwerfelli

College of Dental Medicine, QU Health, Qatar University, Doha, Qatar

E-mail: melwerfelli@qu.edu.qa

Online questionnaire

Demographics

* Required

Q1. Name of the dental institution/university? *

Q2. Location of the dental institution/university? *

Mark only one oval.

- Kingdom of Bahrain
- Kingdom of Saudi Arabia
- State of Kuwait
- Sultanate of Oman
- State of Qatar
- United Arab Emirates

Q3. Type of dental institution/university? *

Mark only one oval.

- Public (Government)
- Private
- Hybrid

Q4. Which undergraduate dental degree does your dental institution/university offer? *

Mark only one oval.

- 5-year Bachelor of Dental Surgery (BDS)
- 5-year Doctor of Dental Medicine (DDM)
- 5-year Doctor of Dental Surgery (DDS)
- 6-year Bachelor of Dental Surgery (BDS)
- 6-year Doctor of Dental Medicine (DDM)
- 6-year Doctor of Dental Surgery (DDS)
- Other:

Q5. What is the average number of dental students who graduate/will graduate each year from your dental institution/university? *

Mark only one oval.

- 1-25 students
- 26-50 students
- 51-75 students
- 76-100 students
- 101-150 students
- 151-200 students
- More than 200 students
- Other: ______________________

Q6. Do you use VRDS during pre-clinical training of your undergraduate dental students? *

Mark only one oval.

- No *Skip to question 7*
- Yes *Skip to question 9*

Dental virtual simulators are not currently present in your university

Q7. Is your institution/university considering the purchase of VRDS in the near future? *

Mark only one oval.

- Yes
- No

Q8. Identify the main reasons why VRDS is not used at your institution/university? *

Check all that apply.

- [ ] Initial upfront cost
- [ ] Maintenance cost
- [ ] Availability of experienced staff
- [ ] Time to practice
- [ ] Other: ______________________

Dental virtual simulators are used in your college

Q9. Which VRDS system is being used at your institution/university? *

Check all that apply.

- [ ] CDS-100 (EPED Inc.)
- [ ] DentSim™
- [ ] Individual Dental Education Assistant (IDEA)
- [ ] Moog Simodont Dental Trainer
- [ ] PerioSim®
- [ ] SIMtoCARE
- [ ] Virteasy Dental
- [ ] Voxel Man
- [ ] Other: ______________________

Q10. Please provide details of VRDS teaching in various courses/modules at your institution?

Check all that apply.

	Not used	Year 1	Year 2	Year 3	Year 4	Year 5	Year 6
**Manual dexterity**	☐	☐	☐	☐	☐	☐	☐
**Dental charting**	☐	☐	☐	☐	☐	☐	☐
**Conservative/Restorative/Operative Dentistry**	☐	☐	☐	☐	☐	☐	☐
**Endodontics**	☐	☐	☐	☐	☐	☐	☐
**Implantology**	☐	☐	☐	☐	☐	☐	☐
**Oral Surgery (Inc. Local Anesthesia)**	☐	☐	☐	☐	☐	☐	☐
**Orthodontics**	☐	☐	☐	☐	☐	☐	☐
**Pediatric Dentistry**	☐	☐	☐	☐	☐	☐	☐
**Periodontics**	☐	☐	☐	☐	☐	☐	☐
**Prosthodontics**	☐	☐	☐	☐	☐	☐	☐
**Other**	☐	☐	☐	☐	☐	☐	☐

If Other was selected above, please specify

____

Q11. What are the average number of contact hours for VRDS training in individual courses/modules?

Check all that apply.

	Not applicable	1-10 hours	11-20 hours	21-30 hours	31-40 hours	above 40 hours
**Manual dexterity**	☐	☐	☐	☐	☐	☐
**Dental charting**	☐	☐	☐	☐	☐	☐
**Conservative/Restorative/Operative Dentistry**	☐	☐	☐	☐	☐	☐
**Endodontics**	☐	☐	☐	☐	☐	☐
**Implantology**	☐	☐	☐	☐	☐	☐
**Oral Surgery (Inc. Local Anesthesia)**	☐	☐	☐	☐	☐	☐
**Orthodontics**	☐	☐	☐	☐	☐	☐
**Pediatric Dentistry**	☐	☐	☐	☐	☐	☐
**Periodontics**	☐	☐	☐	☐	☐	☐
**Prosthodontics**	☐	☐	☐	☐	☐	☐
**Other**	☐	☐	☐	☐	☐	☐

If Other was selected above, please specify

____

Q12. How is VRDS training evaluated? *

Check all that apply.

- [ ] No evaluation is done
- [ ] Automated feedback by simulator software
- [ ] Self evaluation by student
- [ ] Formative feedback by tutor
- [ ] Summative assessment

Q13. In your opinion, what are the benefits of VRDS on the learning experiences of students? *

Check all that apply.

- [ ] Safe learning environment
- [ ] Active hands-on learning
- [ ] Acquisition of technical skills
- [ ] Self-directed learning
- [ ] Reduced need for supervision
- [ ] Opportunities for repeated practice
- [ ] Other: ____________________

Q14. Identify the challenges you have faced with the use of VRDS at your institution/university? *

Check all that apply.

- [ ] Cost of maintenance
- [ ] Availability of experienced staff
- [ ] Limited number of units available
- [ ] Limited time for the students to practice
- [ ] Limited number of tasks available on VRDS library
- [ ] Other: ____________________

Q15. In your opinion, what are the key strengths of VRDS in undergraduate dental education?

Q16. In your opinion, what are the main limitations of VRDS in undergraduate dental education?
